# One-Step Synergistic Treatment Approach for High Performance Amorphous InGaZnO Thin-Film Transistors Fabricated at Room Temperature

**DOI:** 10.3390/nano12193481

**Published:** 2022-10-05

**Authors:** Chunlan Wang, Yuqing Li, Yebo Jin, Gangying Guo, Yongle Song, Hao Huang, Han He, Aolin Wang

**Affiliations:** 1 School of Science, Xi’an Polytechnic University, Xi’an 710048, China; 2 Guangxi Key Laboratory of Processing for Nonferrous Metals and Featured Material, School of Resources, Environment and Materials, Guangxi University, Nanning 530004, China

**Keywords:** a-InGaZnO, TFTs, oxygen plasma, electrical properties

## Abstract

Amorphous InGaZnO (a-InGaZnO) is currently the most prominent oxide semiconductor complement to low-temperature polysilicon for thin-film transistor (TFT) applications in next-generation displays. However, balancing the transmission performance and low-temperature deposition is the primary obstacle in the application of a-InGaZnO TFTs in the field of ultra-high resolution optoelectronic display. Here, we report that a-InGaZnO:O TFT prepared at room temperature has high transport performance, manipulating oxygen vacancy (V_O_) defects through an oxygen-doped a-InGaZnO framework. The main electrical properties of a-InGaZnO:O TFTs included high field-effect mobility (µ_FE_) of 28 cm^2^/V s, a threshold voltage (V_th_) of 0.9 V, a subthreshold swing (SS) of 0.9 V/dec, and a current switching ratio (I_on_/I_off_) of 10^7^; significant improvements over a-InGaZnO TFTs without oxygen plasma. A possible reason for this is that appropriate oxygen plasma treatment and room temperature preparation technology jointly play a role in improving the electrical performance of a-InGaZnO TFTs, which could not only increase carrier concentration, but also reduce the channel-layer surface defects and interface trap density of a-InGaZnO TFTs. These provides a powerful way to synergistically boost the transport performance of oxide TFTs fabricated at room temperature.

## 1. Introduction

High-performance TFTs are the core components of large area electronic devices, which have important research significance in the field of flexible flat panel displays and portable flexible electronic devices [1,2,3]. Metal-oxide semiconductor TFTs are less restricted than amorphous silicon, low temperature polysilicon, and organic TFTs in terms of processing temperatures and fabrication techniques [4,5,6,7]. Among the various metal-oxide TFTs, transparent a-InGaZnO TFTs have been widely studied due to their excellent optical and electrical properties for potential applications in flexible electronics, sensors, and memory devices [8,9,10]. However, in order to further realize the application of a-InGaZnO TFTs in new photoelectric technology, the electrical performance (such as µ_FE_, I_on_/I_off_, V_th_, SS, etc.) of a-InGaZnO TFTs should be improved.

The oxygen-vacancy defect in engineering has always been an important concern and contact point in the study of the electrical properties of a-InGaZnO TFTs [11,12,13,14]. In order to obtain high-performance a-InGaZnO TFTs, high-temperature inert atmosphere, or high-purity oxygen, thermal annealing is usually required; however, this greatly limits their application in the field of flexible and transparent electronics. Therefore, developing high-performance a-InGaZnO TFTs at low temperatures (RT ~ 200 °C), with a large area, is a primary obstacle to application in new electronics. Magnetron sputtering is a commonly used technologies for large-scale industrial production of a-InGaZnO TFTs. Controlling the sputtering power, temperature, sputtering thickness, carrier gas, and other parameters can directly affect the quality of a-InGaZnO, so as to achieve effective regulation of the electrical properties of deposited a-InGaZnO films. At the same time, magnetron sputtering technology can also be used to select the contact electrodes of sputtering TFTs devices [15,16]. The plasma treatment method is an effective method of improving the defects of thin films which simplifies the device structure and improves the device characteristics [11,17,18]. However, the obtained channel layer thickness was relatively thicker, it required high-temperature annealing (more than 250 °C), and the electrical performance needed to be further improved.

Inspired by this success, oxygen plasma treatment was carried out on the basis of ensuring that the whole experimental preparation process of a-InGaZnO TFT was carried out at room temperature. The details were described as follows: first of all, determining a moderate sputtering power controlled the film formation rate of a-InGaZnO film and obtained a relatively high-quality channel-layer film, in order to provide certain high-performance a-InGaZnO TFT parameters, such as good µ_FE_, a V_th_ close to 0 V, relatively large I_on_/I_off_, small SS, etc. This also paved the way for easy control and reduction of channel-layer thickness later. Then, by reducing the sputtering time, under the appropriate sputtering power, the thickness of the a-InGaZnO film was gradually decreased. So, an a-InGaZnO film with small film thickness, good quality, and good comprehensive performance was obtained. After that, a one-step oxygen-plasma treatment method optimized the a-InGaZnO film obtained in the above step. An a-InGaZnO film with high quality and the best comprehensive performance was obtained by regulating different processing power at the same time. Finally, a-InGaZnO:O TFTs were obtained by sputtering copper electrodes with good ohmic contact. In addition, the intrinsic physical mechanism was explained by combining the electrical performance test results and microscopic characterization.

## 2. Experimental Method and Characterization

### 2.1. Device Preparation

**Figure 1:**
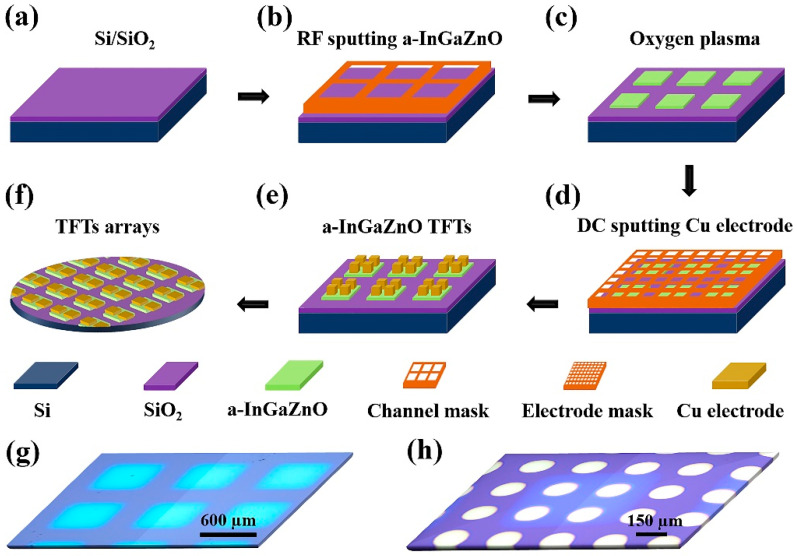
Schematic diagram of the fabrication process of a-InGaZnO/a-InGaZnO:O TFTs: (**a**) Cleaned Si/SiO_2_ substrate; (**b**) RF magnetron sputtering deposition patterned a-InGaZnO channel layer; (**c**) the a-InGaZnO channel layer, treated with oxygen plasma; (**d**) patterned Cu electrodes deposited by DC sputtering; (**e**) patterned a-InGaZnO/a-InGaZnO:O TFTs; (**f**) TFTs arrays; (**g**) Olympus microscope images of channeling layer and (**h**) a-InGaZnO TFTs.

The a-InGaZnO based TFT was pattern-grown by RF magnetron-sputtering technology, as shown in Figure 1. The target material was a high-purity InGaZnO (In:Ga:Zn=1:1:1) ceramic target with a purity of 99.99%. Firstly, in the magnetron sputtering system ((JSD 400, Anhui, China), the target material and Si/SiO_2_ sheltered with channel mask were placed on the target fixed platform and support platform, respectively.

The baffle plate above was closed. The pressure of the sputtering system was closed to below 2 × 10^−4^ Pa, and high-purity Ar was used as the working gas, with a flow rate of 10 SCCM. When the pressure was stabilized, and reached 0.5 Pa, sputtering power could be set to 50 W, 40 W, or 30 W, respectively. After removing impurities on the surface of the target by pre-sputtering, the target baffle and sample baffle were opened, and a-InGaZnO films of 10 nm, 20 nm, and 30 nm were grown by controlling the sputtering time, respectively.

Then, a-InGaZnO channel layers were treated by oxygen plasma, in the plasma system (JSD 200, Anhui, China). The power of oxygen plasma was 10 W, 15 W, 20 W, 30 W, and 40 W, respectively, for 30 s. Finally, a 50 nm Cu electrode was deposited by DC sputtering. The channel length and width of all devices were 120 μm and 150 μm, respectively.

### 2.2. Device Characterization

In this experiment, a Scanning Electron Microscope (SEM; Oxford Anta–450, FEI, Oxford, UK) and an Atomic Force Microscope (AFM, Vista Scope Molecular Vista, San Jose, CA, USA) were used to measure the thickness and surface roughness of the active layer. The thickness of different films was measured by an automatic ellipsometer (TPY-2, Shanghai, China). Transmittance measurements were carried out via a double-beam UV–vis–NIR spectrometer (NOVA2S–EX, Shanghai FuXiang Optics Co. LTD, Shanghai, China). Different a-InGaZnO films were analyzed by X–ray photoelectron spectroscopy (XPS, Thermo ESCALAB 250X, Waltham, MA, USA, C1s revised at 284.8 eV).

The electrical properties of a-InGaZnO TFTs were measured at room temperature using an atmospheric probe station (Optem 70XL, Semishare, HongKong, China) and semiconductor parameter analyzer (Keysight B2912A, Keysight Technologies, Santa Rosa, CA, USA). When measuring the transfer characteristic curve of a-InGaZnO TFTs, the source–drain voltage (V_ds_) was 1 V, and the gate voltage (V_gs_) was from −50 V to 50 V (forward direction) and from 50 V to −50 V (reverse direction) of the scan voltage. When measuring the output characteristic curve of the device, V_ds_ is the scanning voltage from 0 V to 50 V, and V_gs_ is different voltage values from −20 V to 50 V, 10 V/step. Formulas (1) and (2) can be used to calculate the µ_FE_ and SS of the device, respectively [19,20].

(1) $$\mu_{\mathrm{FE}}=\frac{{\mathrm{LdI}}_{\mathrm{ds}}}{{\mathrm{WV}}_{\mathrm{ds}}C_{\mathrm{ox}}{\mathrm{dV}}_{\mathrm{gs}}}$$

(2) $$\mathrm{SS}={\left(\frac{\mathrm{dlog}\left(I_{\mathrm{ds}}\right)}{{\mathrm{dV}}_{\mathrm{gs}}}\right)}^{-1}$$

L and W are the length and width of the device channel, respectively, and C_ox_ is the capacitance per unit area of the device oxide gate insulating layer (SiO_2_, 100 nm).

In general, the SS value of an oxide TFT is closely related to the interface density of trap states (D_it_) between the semiconductor active layer and the gate dielectric layer. D_it_ can be calculated using Formula (3) [20].

(3) $$D_{\mathrm{it}}=\frac{C_{\mathrm{ox}}}{q^{2}}\left(\frac{\mathrm{qSS}}{k_{B}\mathrm{Tln}10}\right)$$

where *q* is the basic charge of the electron, *k*_B_ is the Boltzmann constant, and *T* is the ambient temperature (300 K).

## 3. Results and Discussion

In the process of preparing thin films by magnetron sputtering, different parameters had a crucial impact on the quality and film-formation rate of the obtained thin films, and the film quality played an extremely important role in the performance of the TFT device. Therefore, the specific effects of channel layer, a-InGaZnO film thickness, and sputtering power on the electrical properties of TFT devices were first discussed in this experiment. Then, the effects of treating the a-InGaZnO active layer with oxygen plasma on the photoelectric properties of a-InGaZnO TFT were analyzed.

### 3.1. Different Sputtering Power

**Figure 2:**
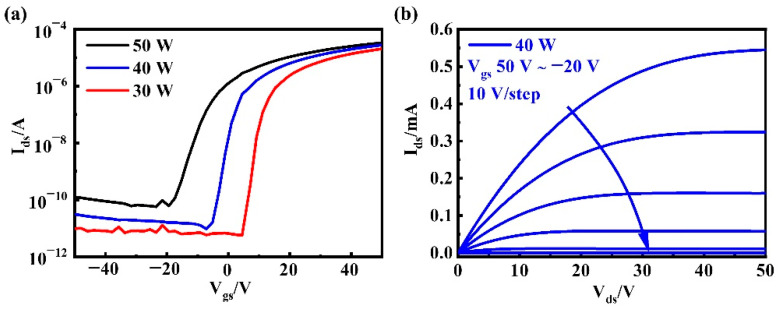
(**a**) Transfer characteristic curves of a-InGaZnO TFTs prepared with different sputtering powers; (**b**) Output characteristic curve of a-InGaZnO TFTs prepared with 40 W sputtering power.

In this experiment, a-InGaZnO TFT was prepared with different sputtering powers (30 W, 40 W, and 50 W) under the same sputtering time (6 min), and the transfer characteristic curves were compared, as shown in Figure 2a. With a decrease in sputtering power, the saturation current of the a-InGaZnO TFT decreased, and V_th_ shifted to the positive direction. Figure 2b shows the output characteristic curve of an a-InGaZnO TFT when the sputtering power was 40 W.

Table 1 shows the main electrical performance of a-InGaZnO TFTs prepared with different sputtering powers. Within a certain range of sputtering power, µ_FE_ and SS decreased with a decrease in sputtering power. With a decrease in sputtering power, the concentration of Zn in the a-InGaZnO film decreased [21]; the concentration of Zn in the film affected the SS of TFTs, such that the SS of the obtained device decreased with the decrease of sputtering power. SS reflects D_it_ to a certain extent, indicating that the density of states of the a-InGaZnO film decreased with the decrease in sputtering power. V_th_ moved in a positive direction with a decrease in sputtering power, indicating that the carrier concentration in the active layer of a-InGaZnO TFTs decreases with a decrease in sputtering power. Therefore, we firstly selected a moderate sputtering power of 40W as a basic parameter to be fixed in the subsequent regulation of channel-layer thickness.

### 3.2. Different Thickness

**Figure 3:**
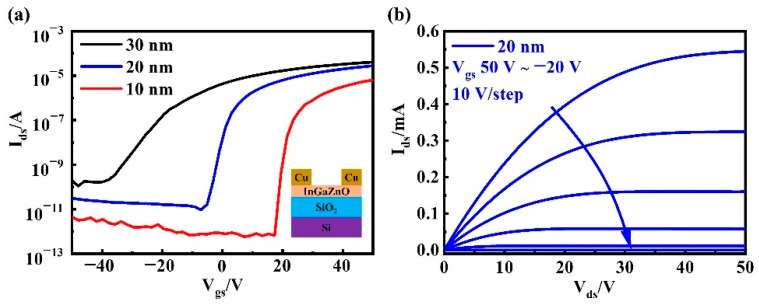
(**a**) Transfer characteristic curves of TFTs with a-InGaZnO active layers of different thicknesses (inset is the cross section of a-InGaZnO TFT); (**b**) Output characteristic curve of a-InGaZnO TFT when the active layer is 20 nm.

Reducing the channel-layer thickness is an effective method of obtaining ultra-thin TFT devices. In this step, a-InGaZnO TFTs with different active-layer thickness (30 nm, 20 nm, and 10 nm) were prepared by controlling sputtering time (9 min, 6 min, and 3 min) under the condition of 40 W sputtering power. Figure 3a shows the transfer characteristic curve of a-InGaZnO TFTs under different active-layer thicknesses. With a decrease in sputtering thickness, the saturation current decreased, and V_th_ displayed a significant positive shift. Obviously, although the saturation current of the a-InGaZnO TFT constructed with the active layer of 20 nm thickness decreased a little, this was still in the same order of magnitude as that of the a-InGaZnO TFT with 30 nm active-layer thickness. This phenomenon was mainly caused by the change of charge carriers, in turn caused by the mass of a-InGaZnO active-layer film. Figure 3b exhibits the output characteristic curve when a-InGaZnO channel thickness was 20 nm.

Table 2 shows the main electrical performance parameters of a-InGaZnO active layer TFTs with different thicknesses. With a decrease in active layer thickness, V_th_ moved in a positive direction, µ_FE_ first increased and then decreased, and SS gradually decreased. When the thickness of the active layer was 10 nm, the D_it_ between the active layer and the gate insulation layer decreased, such that the device had a low SS. However, the interface effect was more obvious at this time, the interface scattering effect was enhanced, and the electron consumption rate in the active layer was increased, such that the µ_FE_ of the device was significantly reduced.

In particular, a 20 nm a-InGaZnO film with 40 W sputtering power was finally selected, as a compromise solution, to construct a-InGaZnO-based TFTs, which were used as the basic control group in the process of this study. These TFTs devices have a low V_th_ (−0.9 V), close to 0 V, an ideal µ_FE_ (17 cm^2^/V s), and a small SS (1.7 V/dec). This is mainly because the trap density in the film decreases with a decrease in thickness, and the carrier scattering and resistance between the contact electrode and channel layer decrease accordingly [22,23,24]. The performance of the a-InGaZnO film deposited with 40 W sputtering power at room temperature shows relatively good film quality and relatively appropriate oxygen defect density, thus providing an a-InGaZnO TFT that has good electrical performance. However, the specific reasons still need subsequent comparative experiments and microscopic characterization.

### 3.3. Oxygen Plasma Treatment

**Figure 4:**
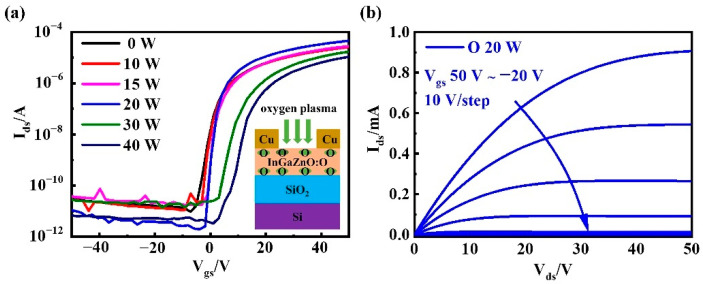
(**a**) Transfer characteristic curves of a-InGaZnO TFTs in the deposition state and a-InGaZnO TFTs in the active layer treated with oxygen plasma of different powers (inset is the cross section of a-InGaZnO:O TFTs); (**b**) Output characteristic curve of TFTs after a-InGaZnO active layer was treated with 20 W oxygen plasma power.

In order to further optimize the electrical performance of a-InGaZnO TFTs, the a-InGaZnO channel-layer films, based on Si/SiO_2_ prepared using the above experimental process, were processed by oxygen plasma with different power (0 W, 10 W, 20 W, 30 W, and 40 W) for 30 s in the plasma system. The electrical properties of TFTs under different conditions were tested. Figure 4a shows the transfer characteristic curves of a-InGaZnO:O TFTs treated by oxygen plasma with different power. With an increase in oxygen plasma power, V_th_ shifted in the positive direction, and the saturation current of the device changed in different amplitude. Figure 4b shows the output characteristic curve of an a-InGaZnO:O TFT treated with 20 W oxygen plasma.

Table 3 shows the electrical parameters of a-InGaZnO:O TFTs treated with differently powered oxygen plasma. V_th_ shifted in the positive direction with an increase in oxygen plasma power. The diffusion of oxygen plasma filled the V_O_ inside the a-InGaZnO semiconductor, thereby reducing its carrier concentration, which in turn led to a positive V_th_ shift [25]. With an increase in oxygen plasma power, the µ_FE_ was increased firstly, and then decreased, which was related to the density of trap states on the channel layer–insulating layer interface, as well as the surface of the channel layer [26,27]. However, high oxygen plasma power will aggravate the surface roughness of the active layer, increase the defect density, hinder the electron movement, and reduce the µ_FE_ [28]. The SS of amorphous oxide TFTs was mainly determined by the presence of deep defects in the active layer and the interface trap-state density between the active layer and the gate-insulating layer [19,29]. The SS value first decreased and then increased with an increase of oxygen plasma power. Excessive oxygen plasma power increases the surface roughness and leads to an increase in defect states, such that the SS value of the device increases. Hence, oxygen plasma treatment at 20 W for 30 s is an optimal parameter for the synergistic improvement of a-InGaZnO TFT preparation at room temperature.

### 3.4. Characterization Analysis of a-InGaZnO Based Channel

**Figure 5:**
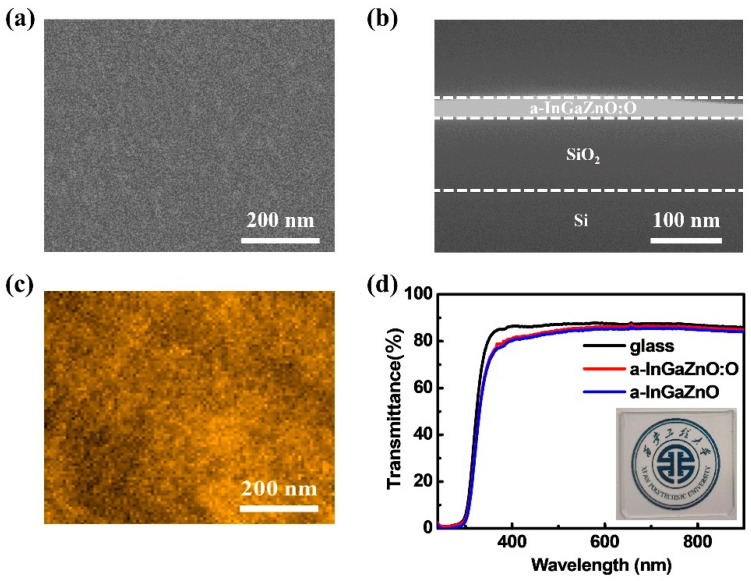
(**a**) SEM images of the surface and (**b**) cross-section of a 20 nm thick a-InGaZnO:O channel layer; (**c**) AFM image of 20 nm thick a-InGaZnO:O channel layer; (**d**) Curves of optical transmittance of glass and a-InGaZnO-based thin film as a function of wavelength. The inset is the photo of an a-InGaZnO:O film (20 nm) on the school emblem.

In order to better explain the mechanism of the effect of oxygen plasma on the performance of a-InGaZnO based TFTs prepared at room temperature, we carried out relevant microscopic characterization. Figure 5a,b shows SEM images of an a-InGaZnO:O film surface and cross section, respectively. The a-InGaZnO:O-film thickness is about 20 nm. Figure 5c shows a surface AFM image of a 20 nm a-InGaZnO:O film; the root mean square roughness is 0.6 nm. The main source of a-InGaZnO:O-film roughness is the atomic mean fluctuation of deposition process statistics. Figure 5d shows the function curve of the optical transmittance of glass and a-InGaZnO-based films as a function of wavelength, which remained above 80% in visible light and higher wavelength regions. The inset is a photo of an a-InGaZnO:O film deposited on a glass substrate, which is overlaid on the school emblem. The results show that the light transmittance of the films treated with oxygen plasma is improved, which is due to a reduction in light scattering and improvement of surface uniformity.

In general, the results show that the light transmittance of films treated with oxygen plasma is improved, which is due to a reduction of light scattering and improvement of surface uniformity. These morphological characteristics show that a-InGaZnO:O film has high surface flatness, which makes its interface with source leakage pole and gate dielectric layer have good interface characteristics, and thus provides necessary support for the subsequent construction of TFTs devices and the acquisition of high performance.

**Figure 6:**
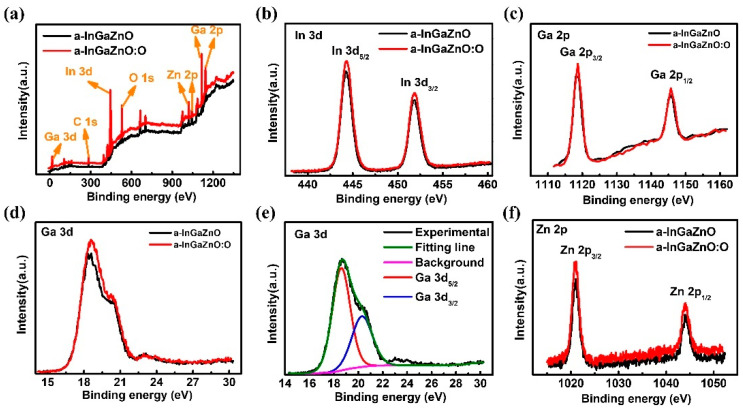
XPS spectra of different elements in a-InGaZnO based film: (**a**) full spectrum; (**b**) In 3d; (**c**) Ga 2p; (**d**) Ga 3d; (**e**) analytical images of Ga 3d peaks; (**f**) Zn 2p.

In addition, combined with microstructure characterization, it is very meaningful and necessary to further determine the mechanism of the influence of oxygen plasma treatment on the electrical properties of a-InGaZnO TFTs prepared at room temperature. XPS measurements were performed on deposited a-InGaZnO films, as well as on a-InGaZnO:O films treated with 20 W oxygen plasma. Figure 6a shows the typical XPS full spectrum of a-InGaZnO and a-InGaZnO:O films. There are no other impurity peaks in the whole spectrum. As shown in Figure 6b, the In 3d_5/2_ and In 3d_3/2_ peaks in a-InGaZnO and a-InGaZnO:O films were located at 444.3 eV and 451.8 eV, respectively. Figure 6c shows the Ga 2p_3/2_ and Ga 2p_1/2_ peaks in a-InGaZnO based films, with peak positions at 1118.7 eV and 1144.1 eV, respectively. The Ga 3d in a-InGaZnO based films was located at 18.7 eV, as shown in Figure 6d. The Ga 3d peak was analyzed and de-convoluted to two peaks, one located at 18.7 eV, from Ga 3d_5/2_ state, and the other located at 20.3 eV from Ga 3d_3/2_ state, as shown in Figure 6e. Figure 6f shows the XPS spectra of Zn 2p_3/2_ and Zn 2p_1/2_ in a-InGaZnO based films, and their peak positions were located at 1021.3 eV and 1044.2 eV, respectively. Based on the above results, the chemical bounding states were not changed by oxygen plasma.

**Figure 7:**
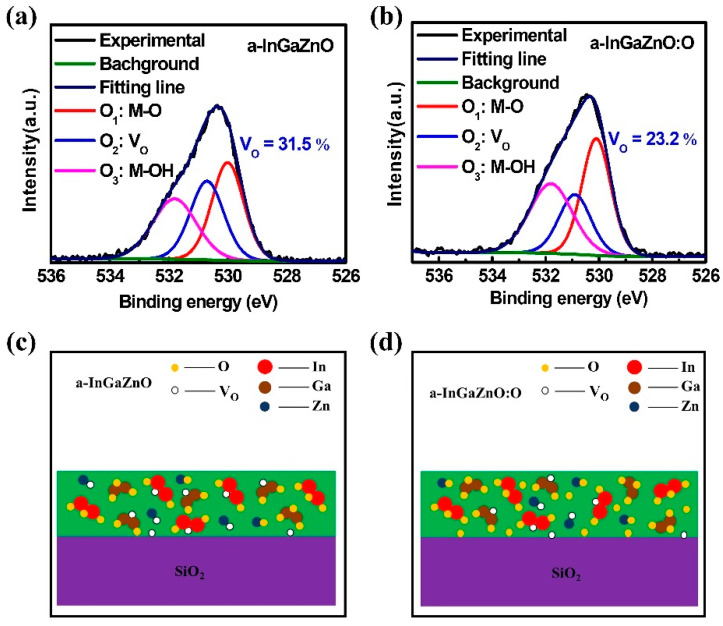
XPS spectra of O 1s in (**a**) a-InGaZnO film and (**b**) a-InGaZnO:O film; Schematic diagram of V_O_ changes in (**c**) a-InGaZnO film and (**d**) a-InGaZnO:O film.

Therefore, with the help of XPS spectrum, we analyzed the V_O_ changes in a-InGaZnO films before and after oxygen plasma treatment, as shown in Figure 7a,b. The measured O 1s spectrum can be divided into three different peaks; the binding energies of the three peaks are O_1_: 529.91 eV, O_2_: 531 eV, and O_3_: 531.8 eV, where the O_1_ peak represents the bonds formed by metal ions and O ions (M-O), such as In-O, Ga-O, and Zn-O [30]; O_2_ peaks indicate defect states formed by V_O_ in films [31,32]; and O_3_ peaks represent the bonds (M-OH) formed between metal ions in the film and some species on the sample surface, such as –CO_3_, OH^–^, and H_2_O [32]. As is shown in Figure 7a,b, the V_O_ of 20 nm a-InGaZnO films deposited by sputtering at 40 W at room temperature is 31.5%, while the V_O_ of a-InGaZnO:O films treated by 20 W oxygen plasma for 30 s is decreased to 23.2 %. It was also confirmed that 20 W oxygen plasma treatment for 30 s has the best effect on synergistic improvement of the quality and TFT electrical properties of 20 nm InGaZnO films deposited by 40 W sputtering at room temperature.

The schematic diagram of V_O_ changes in different films is shown in Figure 7c,d. Although V_O_ functions as an electron donor in the a-InGaZnO channel layer film, most V_O_ are unstable deep defect states, and only a small fraction of V_O_ are located near the conduction band minimum [33,34]. Oxygen plasma, with appropriate power, can replace the traditional thermal annealing treatment, reduce the V_O_ in a-InGaZnO films prepared at room temperature, improve the quality of the channel layer, optimize the interface between the channel layer and gate dielectric layer, and optimize the interface between the channel layer and the contact electrode. Therefore, the electrical properties of a-InGaZnO films prepared at room temperature are improved by oxygen plasma treatment.

Subsequently, we compared this work with related reports on the main sputtering process and electrical properties of a-InGaZnO-based TFTs, as shown in Table 4. In this work, 20 W oxygen plasma treatment for 30s had the best effect on synergistic improvement of the quality and TFT electrical properties of 20 nm InGaZnO films deposited by 40 W sputtering at room temperature. The high-performance InGaZnO TFT device showed high µ_FE_, V_th_ close to 0 V, and low SS. This also shows that an a-InGaZnO film sputtered at 40 W for 6 min can obtain relatively high properties. In 20 nm a-InGaZnO TFTs at room temperature, oxygen ion treatment at 20 W for 30 s has an obvious re-optimization effect on the performance of the above devices. Therefore, a high-performance a-InGaZnO TFT at room temperature needs the joint effect of sputtering thickness, sputtering power, and one-step oxygen plasma treatment.

## 4. Conclusions

In summary, based on systematic oxygen-vacancy defect engineering, a simple channel structure was developed to synergistically improve the transport performance of a-InGaZnO TFTs prepared at room temperature. Firstly, in the process of magnetron sputtering, an a-InGaZnO TFT with certain performance fabricated at room temperature was obtained by coordinating the optimal sputtering power (40 W) and minimum a-InGaZnO film thickness (20 nm), after which a copper source/drain with good ohmic contact was deposited. This device had μ_FE_ of 17 cm^2^/V s, V_th_ of −0.9 V, SS of 1.7 V/dec, and I_on_/I_off_ of 10^6^. The electrical properties of a-InGaZnO films were then optimized by simple and effective oxygen plasma treatment, such that V_th_ of 0.9 V, μ_FE_ of 28 cm^2^/V s, SS of 0.9 V/dec, and I_on_/I_off_ of 10^7^ were obtained. According to the test results and microscopic characterization, the main underlying reason for the enhanced electrical properties of a-InGaZnO TFTs is the appropriate oxygen plasma treatment process and room temperature preparation technology, which jointly play a role in improving the electrical performance of a-InGaZnO TFTs. In other words, appropriate oxygen defect density can not only increase the carrier concentration of an a-InGaZnO channel layer deposited at room temperature, but also reduce the surface defects and interface trap density of a-InGaZnO TFTs. Hence, our method of adjusting the oxygen defect density in devices prepared at room temperature provides an effective and low-cost method for the application of a new generation of oxide optoelectronic devices and systems.

## Figures and Tables

**Table 1:** Electrical parameters of a-InGaZnO TFTs prepared with different sputtering powers.

RF Power (W)	V_th_ (V)	µ_FE_ (cm^2^/V s)	I_on_/I_off_	SS (V/dec)	D_it_ (cm^−2^/eV)
50	−10	18	10^5^	2.9	1.1 × 10^11^
40	−0.9	17	10^6^	1.7	6.3 × 10^10^
30	5	14	10^6^	1.2	4.5 × 10^10^

**Table 2:** Electrical parameters of a-InGaZnO TFTs with different channel thicknesses.

Channel Thickness (nm)	V_th_ (V)	µ_FE_ (cm^2^/V s)	I_on_/I_off_	SS (V/dec)	D_it_ (cm^−2^/eV)
30	−19	14	10^5^	5.6	2.1 × 10^11^
20	−0.9	17	10^6^	1.7	6.3 × 10^10^
10	19	6	10^6^	0.7	2.6 × 10^10^

**Table 3:** Electrical parameters of a-InGaZnO:O TFTs with differently powered oxygen plasma.

Oxygen Power (W)	V_th_ (V)	µ_FE_ (cm^2^/V s)	I_on_/I_off_	SS (V/dec)	D_it_ (cm^−2^/eV)
0	−0.9	17	10^6^	1.7	6.3 × 10^10^
10	−0.8	18	10^6^	1.5	5.7 × 10^10^
15	0.3	19	10^6^	1.3	4.8 × 10^10^
20	0.9	28	10^7^	0.9	3.3 × 10^10^
30	9.1	14	10^5^	1.9	7.1 × 10^10^
40	12	10	10^6^	2.2	8.2 × 10^10^

**Table 4:** Comparison of preparation parameters and electrical properties of various a-InGaZnO TFT deposited by sputtering.

RF Power (W)	Channel Thickness (nm)	Plasma	Annealing Temperature (°C)	Contact Electrode	µ_FE_ (cm^2^/V s)	I_on_/I_off_	V_th_ (V)	SS (V/dec)	Ref.
40	20	– oxygen	RT	Cu	17 28	10^6^ 10^7^	−0.9 0.9	1.7 0.9	This work
/	50	– oxygen	120	Ti/Au	14.4 16.9	10^6^ 10^5^	−23 4.3	0.6 1.3	[35]
/	15	– oxygen	250	Al	7.3 10.1	10^7^ 10^7^	1.4 0.5	0.3 0.2	[11]
150	50	– oxygen	300	Al	8.9 14.8	10^2^ 10^8^	5.1 0.4	7.3 0.6	[28]
/	50	–	300	Al	5.7 9.6	10^7^ 10^8^	5.3 8.4	1.1 0.5	[12]
150	50	– oxygen	350	Al	10.6 14.4	10^7^ 10^8^	2.8 4.5	0.9 0.7	[13]
/	30	–	350	Ti/Au	4.8	10^7^	9.1	1.2	[15]
30	50	–	350	/	7.8	10^7^	12.7	1.1	[16]

## Data Availability

The data presented in this study are available on request from the corresponding author. The data are not publicly available due to privacy.
